# Study on the aging performance of SBS modified asphalt in humid and hot environment

**DOI:** 10.1371/journal.pone.0325103

**Published:** 2025-06-16

**Authors:** Xiaoyan Liu, Ping Li, Jingsheng Pan

**Affiliations:** Department of Civil Engineering, Chengdu Technological University, Yibin, China; Oregon State University, UNITED STATES OF AMERICA

## Abstract

This study focused on the aging issues of asphalt pavement in high-temperature and high-humidity climatic zones, using SBS-modified asphalt with different additive contents as research objects to systematically investigate the aging evolution mechanisms under coupled hygrothermal conditions. A dual-temperature control system (60°C and 70°C) was constructed to conduct thermal-oxidative aging and wet-dry cycle aging tests. Multidimensional indicators including penetration ratio, softening point ratio, and ductility ratio were utilized to characterize the time-dependent aging characteristics of materials. Additionally, dynamic shear rheology (DSR) tests and rutting tests were integrated to comprehensively evaluate the high-temperature performance degradation of asphalt and its mixtures with varying modifier contents under cyclic aging. The research aimed to reveal the hygrothermal-coupled aging mechanisms and provide theoretical foundations for durable pavement design in humid tropical regions. Experimental results demonstrated that after thermal-oxidative and wet-dry cycle aging at 60°C and 70°C, the performance indices of SBS-modified asphalt with different additive contents deteriorated significantly. Specifically, the penetration ratio decreased by an average of 29.1%, the ductility ratio declined by 14.6%, and the softening point ratio increased by 60.3%. Concurrently, high-temperature rheological performance exhibited progressive degradation. Notably, increased SBS modifier content was observed to mitigate the aging-induced performance attenuation of modified asphalt. Analytical findings suggested that while higher SBS content enhanced structural stability to some extent, the presence of elevated temperature and moisture accelerated the degradation of SBS modifiers and the aging of base asphalt, thereby exacerbating the overall aging of modified asphalt and leading to performance deterioration.

## 1. Introduction

Asphalt pavement has been extensively utilized in high-grade highway construction due to its superior service performance. However, during service life, asphalt is continuously subjected to coupled environmental factors including heat, oxygen, ultraviolet (UV) radiation, and moisture [1–3], leading to significant physicochemical deterioration that manifests as pavement distresses such as cracking and raveling, ultimately resulting in severe lifespan reduction [4,5]. Particularly in hot and humid regions, the synergistic effects of elevated temperature and high humidity accelerate asphalt aging processes, rendering the aging mechanisms more complex. Therefore, investigating the aging behavior and mechanisms of SBS-modified asphalt under hygrothermal conditions holds significant theoretical and engineering importance for enhancing the durability of pavement materials.

Polymer modified asphalt is one of the best choices to improve the aging resistance and physical properties of asphalt [6]. Because of its unique performance, SBS can effectively improve the physical and rheological properties of asphalt, reduce the temperature sensitivity to a certain extent, and improve the rutting resistance, crack resistance and durability [7–9], so it is widely used in asphalt and asphalt mixtures. However, due to the oxidation of asphalt and degradation of SBS copolymers, SBS modified asphalt ages, resulting in increased temperature sensitivity and severe water damage [10–13]. More and more scholars are paying attention to the changes in performance of SBS modified asphalt before and after aging. At present, research on SBS modified asphalt aging mainly focuses on quantitative evaluation of aging mechanisms and aging reaction chains, with a wide range of research scope.Islam et al. [14] stored SBS asphalt at 180°C for 7 days, resulting in a 33% decrease in Marshall stability and a 50% increase in rut depth of the prepared asphalt mixture. Tan et al. [15] proposed five quantitative parameters and used multiple logistic regression analysis to classify the phase structure that increases with aging time. By evaluating its toughness curve, the failure of the modification effect was determined, and this was used as a parameter to determine whether the aged asphalt was modified asphalt or matrix asphalt. Xiong et al. [16] incorporated Mxene (Ti3C2Tx) into SBS modified asphalt based on the characteristics of SBS copolymer degradation and asphalt photooxidation, improving its resistance to UV aging and rutting. Gao et al. [17] aged SBS modified asphalt in different ways and found that after long-term aging, the SBS copolymer lost its modification function. Zhang et al. [18] studied the structural changes of SBS copolymers before and after aging of SBS modified asphalt. The study showed that the structural characteristics of SBS copolymers have a significant impact on the rheological properties of SBS modified asphalt before and after aging. Zeng [19] et al. found that adding oxygen resistant, heat resistant and ultraviolet resistant polyolefin elastomer (POE) to SBS modified asphalt can significantly improve the high-temperature stability of asphalt, and the aging resistance of POE/SBS modified asphalt is better than that of SBS modified asphalt. Zhang et al. [20] analyzed the correlation and differences in rheological and microstructural properties of SBS modified asphalt under field aging and laboratory aging. Xu et al. [21] studied the aging behavior of SBS modified asphalt with different SBS contents before and after aging by testing the aging mechanism, conventional properties, rheological properties, and chemical structure of SBS modified asphalt at different degrees. They established a prediction model for the aging behavior of SBS modified asphalt using partial least squares method. Ayman et al. [22] believe that SBS modified asphalt significantly improves its resistance to rutting and durability. Chen et al. [23] studied the effects of multiple cyclic aging and regeneration aging on the rheological, chemical, and morphological properties of SBS modified asphalt. Song et al. [24] and Kabir et al. [25] studied the evolution of natural aging of SBS modified asphalt in complex and harsh climate environments in cold and arid regions based on different environmental factors. They believed that thermal oxidative aging had a more significant impact on the natural aging of SBS modified asphalt. Cao et al. [26] analyzed from the aspects of chemical structure, molecular weight, and performance that the aging of SBS modified asphalt has a dominant impact on its properties during the aging process.

Due to the high cost of SBS, many scholars have attempted to composite industrial waste or other materials with SBS modified asphalt. Hao et al. [27] added waste polyurethane (WPU) particles to SBS modified asphalt and found through microstructure and rheological performance tests that although WPU particles can improve the low-temperature crack resistance of asphalt, they affect the bonding performance of SBS modified asphalt. Wu et al. [28] studied the rheological properties of SBS/CRP composite modified asphalt after aging under low temperature, strong radiation, and heavy load conditions by simulating different aging conditions (as above). Li et al. [29] found that CeO_2_ can effectively increase the decomposition temperature of SBS modified asphalt, improve the thermal stability of SBS modified asphalt, and enhance its resistance to thermal oxidative aging. Vural B K et al. [30] combined solid single sheet photopolymer printing plates (SSP) and SBS to jointly modify asphalt, which also has better high and low temperature stability, providing an economical and environmentally friendly solution without affecting performance. Korzay et al. [31] modified asphalt with solubilizers hydrated lime (HL) and diatomaceous earth in combination with SBS. The results showed that this method can effectively improve the water damage and rutting resistance of asphalt mixtures. Xie et al. [32] studied the influence of aging on the microstructure and rheological properties of graphene and SBS composite modified asphalt. The results show that the addition of graphene not only improves the high-temperature stability of asphalt mixture, but also has better anti-aging performance. Pandey et al. [33] found that the anti rutting performance was improved by 20–40% after using several thermoplastic and SBS modified asphalt.

In summary, the aging of SBS-modified asphalt constitutes an exceptionally complex process. Current research on SBS-modified asphalt aging has primarily focused on investigating its rheological properties, chemical structure, molecular weight, and performance under different aging methods [34–39], as well as composite modifications combining SBS with other materials. However, diverse environments entail distinct aging patterns. China’s vast territory encompasses significant climatic variations, and the influence of natural factors on asphalt performance cannot be overlooked. Consequently, aging mechanisms of SBS-modified asphalt vary substantially across regions and environmental conditions. Although existing studies have thoroughly explored the mechanisms of individual aging factors (e.g., heat, UV, moisture), the following critical gaps persist regarding composite aging behaviors under multi-factor synergies in hot and humid regions: (1) the long-term impact of high-temperature and high-humidity environments on the compatibility between SBS modifiers and asphalt remains unclear; (2) systematic investigations into the coupling effects of UV radiation and moisture infiltration are lacking; (3) the mechanisms underlying colloidal structural reorganization of asphalt under microscale composite aging require further elucidation.

To address these limitations, this study evaluated the pre- and post-aging performance of SBS-modified asphalt under high-temperature and high-humidity conditions, incorporating diverse aging modes. By simulating thermo-oxidative and moisture-coupled aging environments and employing multi-scale analytical techniques—including dynamic shear rheometer (DSR) tests and rutting tests—the research systematically investigated the aging behavior of SBS-modified asphalt in hygrothermal environments. The work aimed to elucidate aging mechanisms driven by multi-factor synergies, thereby providing theoretical support for the design of asphalt pavement materials and the development of anti-aging technologies in hot and humid regions.

## 2. Materials and testing methods

### 2.1 Test materials

The two raw materials selected for this study are road asphalt (SK-70A #) and SBS (1301) particles. Among them, SBS has a linear structure with a relative molecular weight of 100978 and a styrene content of 30.0%. SBS modified asphalt with SBS content of 3.5%, 4.0%, 4.5%, and 5.0% was prepared using these two raw materials. The properties of the base asphalt and SBS modified asphalt with different content are shown in Table 1.

**Table 1:** Performance of Matrix Asphalt and SBS Modified Asphalt.

Types of asphalt	softening point (°C)	Penetration (25°C, 0.1 mm)	Ductility (15°C, cm)	Ductility (5°C, cm)
70#	50.1	64	>150	/
3.5%SBS	57.1	55	/	43.8
4.0%SBS	67.2	57	/	51.7
4.5%SBS	80.3	57	/	47.7
5.0%SBS	84.3	56	/	40.2

### 2.2 Testing methods

In high-temperature and humid regions, SBS modified asphalt is subjected to the coupling effects of moisture, temperature, and oxygen. In this study, the air blowing function of a drying oven was used to dry SBS modified asphalt, and a water bath was used to treat SBS modified asphalt. The actual wet heat aging process of SBS modified asphalt was simulated through wet dry cycles. The dry wet cycle aging in this study was conducted with a 24-hour experimental cycle, in which 10 hours were completely immersed in a water bath to simulate rainwater conditions, and 14 hours were placed in a drying oven at temperatures of 60°C and 70°C. Short term aging (TFOT) was followed by long-term aging at 4, 8, 12, 16, 20, 25, and 30 days. Thermal oxidative aging was simulated using a rotating thin film oven.

The SBS modified asphalt with different dosages used in this study was prepared by heating 70 # asphalt (SK-70A #) to 140°C and adding small amounts of SBS particles in batches on an electric furnace. During this process, the temperature was raised to 160°C, and then the temperature was controlled at 175°C and sheared for 60 minutes under a modification process of 4500r/min.

The performance of aged asphalt is generally represented by penetration ratio (PR), softening point ratio (SPR), and ductility ratio (DRR), where penetration ratio is calculated by formula 2–1, softening point ratio is calculated by formula 2–2, and ductility ratio is calculated by formula 2–3. P_unaged_ and P_aged_ are the penetration values of asphalt before and after aging, SP_unaged_ and SP_aged_ are the softening points of asphalt before and after aging, and D_unaged_ and D_aged_ are the ductility values of asphalt before and after aging. Finally, its high-temperature rheological properties and anti rutting performance were tested through dynamic shear rheological tests and rutting tests.


$$\begin{array}{c} PR=\frac{P_{aged}}{P_{unaged}}\times 100\\ \% \end{array}$$ (1)

$$\begin{array}{c} SPR=\frac{SP_{aged}}{SP_{unaged}}\times 100\% \end{array}$$ (2)

$$\begin{array}{c} DRR=\frac{D_{aged}}{D_{unaged}}\times 100\% \end{array}$$ (3)

## 3. Results and discussion

### 3.1 Effect of aging time on various indicators of SBS modified asphalt

**Fig 1:**
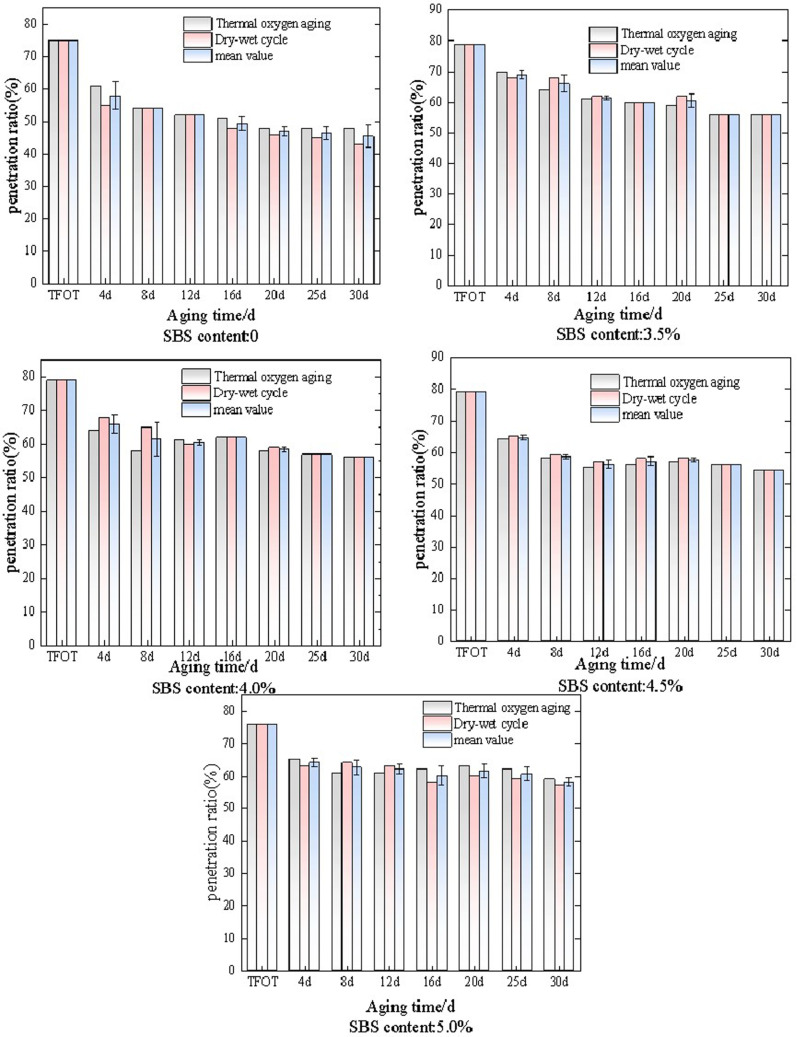
The variation law of needle penetration ratio of SBS modified asphalt under different aging conditions.

After long-term thermal oxidative aging and wet dry cycle aging, the needle penetration, elongation, and softening point were evaluated and analyzed as indicators. SBS modified asphalt with different dosages was subjected to long-term thermal oxidative aging and wet dry cycle aging at 60°C. The aging results were characterized by penetration ratio, and the research results are shown in Fig 1.

As shown in Fig 1, the changes in penetration ratio of SBS modified asphalt with dosages of 0 (matrix asphalt), 3.5%, 4.0%, 4.5%, and 5.0% under long-term thermal oxidative aging and wet dry cycle aging conditions. Overall, as the aging time increases to 30 days, the penetration ratio under both aging methods shows a decreasing trend. Among them, the penetration rate of the matrix asphalt decreased significantly compared to the previous stage. SBS modified asphalt with a dosage of 3.5% gradually decreased during the 30 day aging process due to thermal oxidative aging, and the rate of decrease was faster in the early stage. At the time of short-term aging (TFOT) to 4d, the penetration rate decreased by 11.4%, and at 4d to 8d, the penetration rate decreased by 8.6%, and then gradually slowed down. During this process, the penetration rate decreased by 29.1% overall. The penetration ratio of wet dry cycle decreased by 29.1% overall. It is noteworthy that the penetration ratio exhibited a temporary rebound at 4 days and 8 days of aging, though an overall downward trend was maintained. Analysis suggested that during the initial aging stage (e.g., 4 days), moisture infiltration induced swelling of the SBS phase, which temporarily filled asphalt pores and led to the rebound in penetration values. Conversely, prolonged aging (e.g., 8 days) triggered partial reorganization of polymer chains or redistribution of oxidation products, potentially causing microstructural relaxation that contributed to the periodic fluctuations in penetration ratio.The penetration ratio of SBS modified asphalt with a dosage of 4.0% decreases more rapidly from short-term aging (TFOT) to 4d. Among them, the penetration ratio of thermal oxygen aging decreases by 19.0%, and the penetration ratio of wet dry cycle aging decreases by 11.4%. Moreover, at 4d and 8d, the penetration ratios of the two aging methods differ significantly from other times, at 6% and 12%, respectively. The change in penetration ratio of SBS modified asphalt with a dosage of 4.5% is similar to that of the previous two, and the change rate is also the same. The penetration ratio decreases by 29.1% under both aging methods, but the difference is that the penetration ratio of the two aging methods is not significantly different under the same aging time. When the dosage is 5.0%, the penetration ratio of SBS modified asphalt still shows an overall downward trend, with a decrease of 22.4% under thermal oxidative aging conditions and 25.0% under wet dry cycle aging conditions.

Analysis suggests that as the aging time increases, the aging of SBS modified asphalt gradually intensifies. During this process, the asphalt gradually hardens, the SBS polymer gradually degrades, and the penetration of SBS modified asphalt decreases, ultimately leading to a decrease in penetration ratio. By comparison, the penetration ratio of wet dry cycle aging is more stable than that of thermal oxidative aging, indicating that asphalt is more sensitive to temperature than humidity.

**Fig 2:**
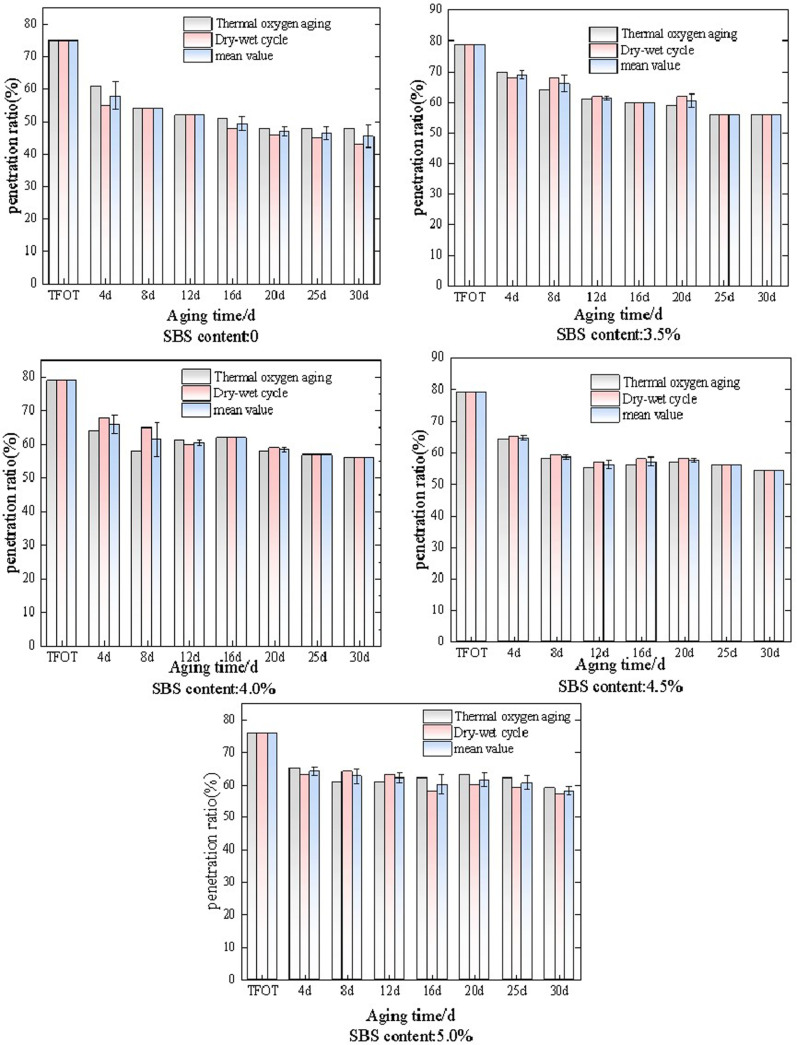
The variation law of softening point ratio of SBS modified asphalt under different aging conditions.

After thermal oxidative aging and wet dry cycle aging, the softening point of SBS modified asphalt with different dosages was tested and represented by the softening point ratio. The changes in softening point ratio under different aging methods and aging times are shown in Fig 2.

As shown in Fig 2, the softening point ratio changes of SBS modified asphalt with dosages of 0 (matrix asphalt), 3.5%, 4.0%, 4.5%, and 5.0% under long-term thermal oxidative aging and wet dry cycle aging conditions. Overall, as the aging time increases to 30 days, the softening point ratio under both aging methods shows an upward trend. Among them, the increase in softening point ratio of matrix asphalt is relatively small. SBS modified asphalt with a dosage of 3.5% gradually increases the softening point ratio during the 30 day aging process due to thermal oxidative aging, and the increase rate is fast in the early stage. When the short-term aging (TFOT) reaches 4d, the softening point ratio increases by 23.2%, and when 4d to 8d, the softening point ratio increases by 4.1%, and then gradually slows down. During this process, the softening point ratio increases by 34.3% compared to the overall. The softening point of the wet dry cycle decreased by 29.3% compared to the overall decrease. It is worth noting that when the aging time was 4 days, the growth rate of the softening point ratio under both aging methods was relatively large, with a 23.2% increase in thermal oxygen aging and a 22.2% increase in wet dry cycle aging, and gradually stabilizing in the later stage. Analytical findings suggested that during the 4-day aging period, both aging methods triggered rapid reorganization of SBS molecular chains and alterations in asphaltene polar components, with their synergistic effects resulting in a sharp increase in the softening point.The SBS modified asphalt with a content of 4.0% showed a relatively slow increase in softening point ratio during short-term aging (TFOT) to 4d. Among them, the softening point ratio of thermal oxygen aging increased by 13.0%, and the softening point ratio of wet dry cycle aging increased by 8.7%. During the aging time periods of 4d and 12d, the softening points of both aging methods showed a decreasing or stable trend, with thermal oxygen aging decreasing by 2.8% and wet dry cycle aging tending to stabilize. The change pattern of the softening point ratio of SBS modified asphalt with a dosage of 4.5% is similar to that of the previous two, except that there is a downward trend in the later stage, and the change rate also decreases. The softening point ratio of the two aging methods only increases by 12.7%, and the difference in softening point ratio between the two aging methods is not significant at the same aging time. When the dosage is 5.0%, the softening point ratio of SBS modified asphalt still shows an overall increasing trend. Among them, the softening point ratio increases by 3.3% under thermal oxidative aging conditions and 5.5% under dry wet cycle aging conditions. It is worth noting that the softening point ratio of SBS modified asphalt with a dosage of 5.0% changes little and tends to be stable.

As the amount of SBS particles added increases, the softening point ratio under both aging conditions gradually decreases, and the rate of change in softening point ratio gradually decreases. Compared with thermal oxidative aging, the softening point ratio under dry wet aging conditions fluctuates more. The analysis shows that the increase of softening point ratio is still caused by the aging of SBS modified asphalt, and the increase of temperature will intensify the degradation of SBS. However, in SBS modified asphalt, the network structure of SBS improves its anti-aging performance, so it is easier to maintain its network structure during aging after the increase of SBS content.

**Fig 3:**
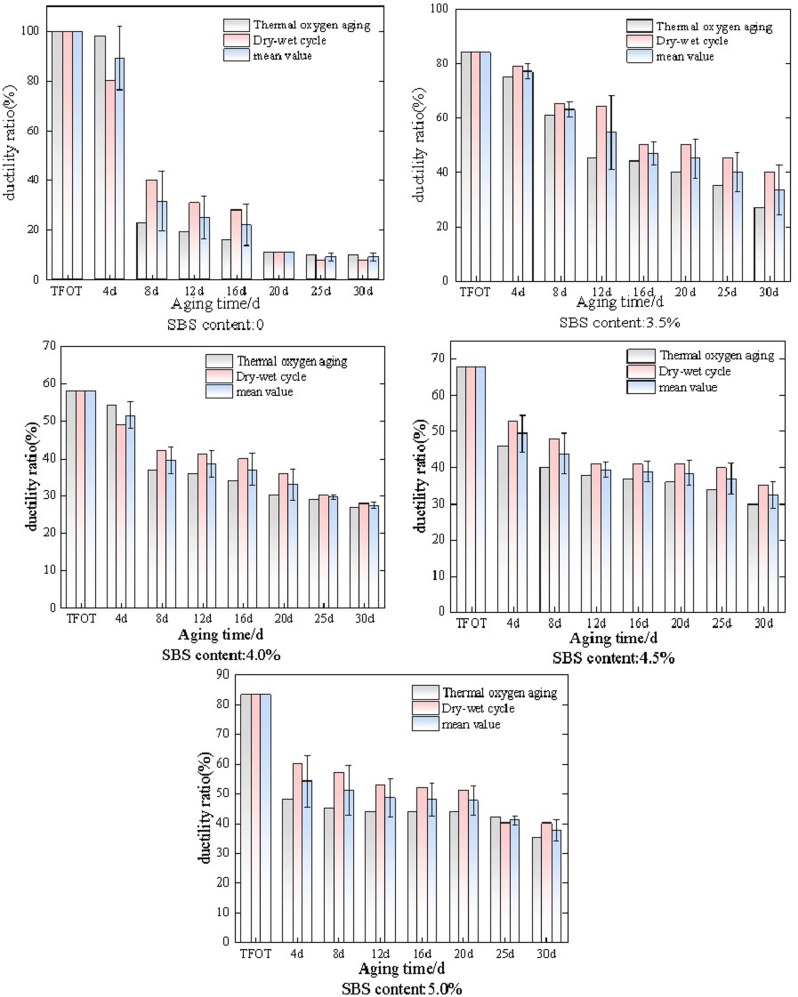
The variation law of SBS modified asphalt ductility ratio under different aging conditions.

The matrix asphalt and SBS modified asphalt with different dosages were subjected to thermal oxidative aging and wet dry cycle aging at 60°C, characterized by the elongation ratio as the index. The changes in elongation ratio under different aging methods and aging times are shown in Fig 3.

As shown in Fig 3, the variation of ductility ratio of SBS modified asphalt with dosages of 0 (matrix asphalt), 3.5%, 4.0%, 4.5%, and 5.0% under long-term thermal oxidative aging and wet dry cycle aging conditions. Overall, as the aging time increases to 30 days, the elongation ratio under both aging modes shows a decreasing trend. Among them, the ductility ratio of the matrix asphalt decreases the fastest. SBS modified asphalt with a dosage of 3.5% gradually decreases during the 30 day aging process due to thermal oxidative aging, and the decrease rate is fast in the early stage, and tends to stabilize in the later stage. When the short-term aging (TFOT) reaches 8 days, the ductility ratio decreases by 27.4%, and during this process, the overall ductility ratio decreases by 67.9%. Under wet dry cycle conditions, the elongation ratio first slowly decreases, then the rate of decrease increases, and finally stabilizes after a rebound, with an overall decrease of 52.4%. It is worth noting that the elongation ratio decreases too quickly, by 17.8%, when the aging time is between 4d and 8d. The change rate of the elongation ratio of SBS modified asphalt with a dosage of 3.5% differs significantly between the two aging methods under the same aging time. Analysis suggests that thermal oxidative aging directly damages the molecular structure of asphalt and SBS, while dry wet cycles are mainly characterized by local physical damage, resulting in a larger rate of change in thermal oxidative aging.The ductility ratio of SBS modified asphalt with a content of 4.0% showed an overall decreasing trend under two aging conditions, and compared with the addition of 3.5% SBS modified asphalt, the ductility ratio fluctuation under the two aging conditions was relatively small. When short-term aging (TFOT) reaches 8 days, the elongation ratio decreases relatively quickly. Among them, the elongation ratio of thermal oxygen aging decreases by 36.2%, and the elongation ratio of dry wet cycle aging decreases by 27.6%. Then, the degree of decrease decreases and eventually stabilizes. The change pattern of the ductility ratio of SBS modified asphalt with a dosage of 4.5% is similar to that of the previous two, except that there is a weak upward trend in the later stage, and the overall change rate has also decreased. The ductility ratio of the two aging methods decreased by 55.9% and 48.5% respectively, and the difference in ductility ratio between the two aging methods is not significant at the same aging time. When the dosage is 5.0%, the softening point ratio of SBS modified asphalt still shows an overall increasing trend. Among them, the ductility ratio decreases by 57.8% under thermal oxidative aging conditions and 51.8% under dry wet cycle aging conditions. It is worth noting that the ductility ratio of SBS modified asphalt with a dosage of 5.0% changes little after short-term aging (TFOT) and tends to stabilize.

Overall, the ductility ratio gradually decreases under both aging conditions. Analysis suggests that long-term aging leads to intensified degradation of SBS copolymers, reducing the ductility of SBS modified asphalt. The change rate of ductility ratio of SBS modified asphalt with the above dosages under dry wet cycle conditions is greater than that under thermal oxidative aging conditions.

### 3.2 The effect of temperature on various indicators of SBS modified asphalt

**Fig 4:**
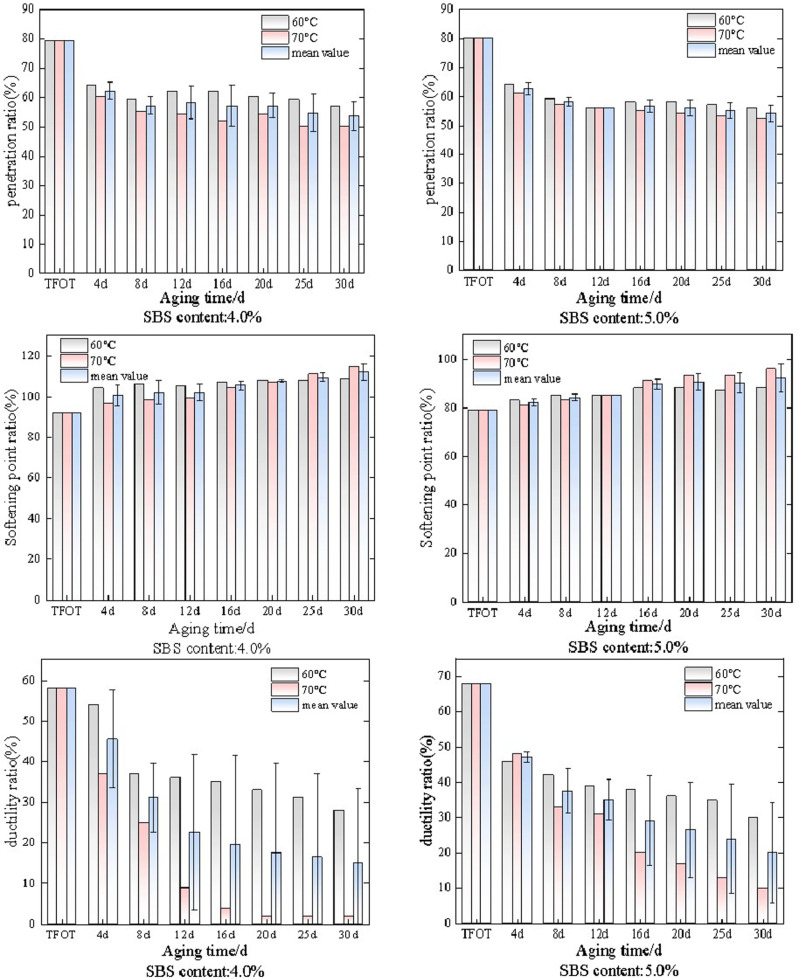
Performance changes of SBS modified asphalt at different temperatures under thermal oxidative aging conditions.

SBS modified asphalt with dosages of 4.0% and 5.0% was selected and subjected to thermal oxidative aging at 60°C and 70°C. The changes in penetration ratio, elongation ratio, and softening point ratio of the two dosages of SBS modified asphalt at different temperatures are shown in Fig 4.

As shown in Fig 4, the changes in penetration ratio, softening point ratio, and ductility ratio of SBS modified asphalt with a dosage of 4.0% and 5.0% after thermal oxidative aging at 60°C and 70°C. Overall, under thermal oxidative aging conditions, as the aging time increases, the penetration ratio and elongation ratio of SBS modified asphalt gradually decrease, while the softening point ratio gradually increases. And under the condition of 70°C, the change rate of the three indicators is greater than that under the condition of 60°C. It is believed that 70 °C accelerates thermal oxidative aging, damages the SBS network structure, and changes the physical state of asphalt, resulting in significantly higher changes in penetration, softening point, and ductility of SBS modified asphalt compared to 60 °C conditions.For the penetration ratio, the two dosages of SBS modified asphalt gradually decreased under the conditions of 60°C and 70°C. However, the penetration ratio of 4.0% SBS modified asphalt fluctuated more at both temperatures than that of 5.0% SBS modified asphalt under the same aging time. Analysis suggests that with the increase of SBS modification, the stability of modified asphalt increases and is relatively less affected by temperature. The penetration ratio of SBS modified asphalt with two different dosages decreased by 29.1%, 36.7%, 29.0%, and 35.0% at different temperatures, respectively. SBS modified asphalt with a dosage of 5.0% was more stable at both temperatures. For the softening point ratio, it is generally increasing. In the early stage, the softening point ratio at 60°C increases rapidly. When the aging time increases to 16 days, the softening point ratio at 70°C increases rapidly. Overall, the softening point ratio at 70°C increases more. The softening point ratios of the two types of SBS modified asphalt at different temperatures increase by 18.5%, 25.1%, 11.4%, and 21.4%, respectively. The softening point ratio of 4.0% SBS modified asphalt at both temperatures fluctuates more than that of 5.0% SBS modified asphalt at the same aging time, indicating that the structure of modified asphalt is relatively stable within a certain range with the increase of SBS content. For the ductility ratio, the difference in ductility ratio between SBS modified asphalt with the same dosage at the same aging time and temperature is significant, and this phenomenon becomes more pronounced with increasing aging time. When the aging time is 4d, the difference between the two dosages of SBS modified asphalt is 31.5% and 11.1%, respectively. When the aging time is 30d, the difference between the two dosages of SBS modified asphalt is 93.1% and 70.0%, respectively.

**Fig 5:**
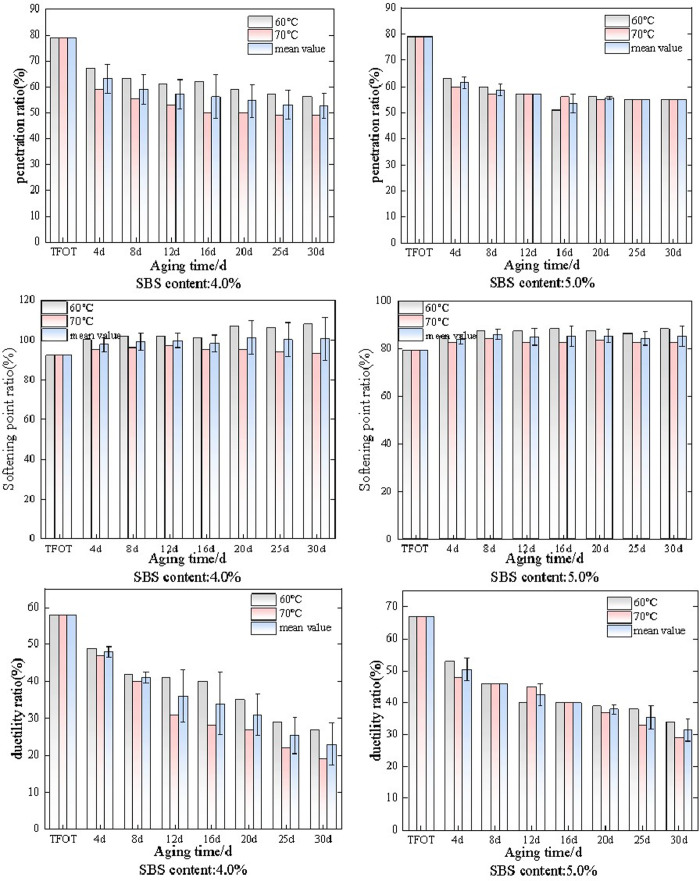
Performance changes of SBS modified asphalt under dry wet cycle aging conditions at different temperatures.

SBS modified asphalt with a dosage of 4.0% and 5.0% was selected and subjected to dry wet cycle aging at 60 °C and 70 °C. The changes in penetration ratio, ductility ratio, and softening point ratio of the two dosages of SBS modified asphalt at different temperatures are shown in Fig 5.

As shown in Fig 5, the changes in penetration ratio, softening point ratio, and ductility ratio of SBS modified asphalt with a dosage of 4.0% and 5.0% after wet dry cycle aging at 60 ° C and 70 ° C. Overall, under the conditions of wet dry cycle aging, as the aging time increases, the penetration ratio and elongation ratio of SBS modified asphalt gradually decrease, while the softening point ratio gradually increases. Moreover, the change rate of the three indicators of SBS modified asphalt with a dosage of 4.0% is greater than that of SBS modified asphalt with a dosage of 5.0%. The analysis shows that 5.0% SBS content forms a more complete network structure and a more stable interface combination, providing a stronger anti-aging capability, so the change rate of its three indicators is less than that of 4.0% SBS modified asphalt.For the penetration ratio, the change rates of the penetration ratio of SBS modified asphalt with a content of 4.0% at two temperatures were 29.8% and 38.0%, respectively, and the change rates of the penetration ratio of SBS modified asphalt with a content of 5.0% at two temperatures were 30.4% and 30.6%, respectively. This indicates that as the content of SBS modified material increases, its volatility decreases, and the difference in the same index under the two temperature conditions decreases. For the softening point ratio, there is little change in the softening point ratio of SBS modified asphalt after wet dry cycle aging at 60 °C and 70 °C. However, the softening point ratio at 60 °C is higher than that at 70 °C, indicating that SBS modified asphalt undergoes more severe degradation under 70 °C wet dry cycle aging. For the ductility ratio, the ductility ratio after wet dry cycle aging at 60 °C is higher than that of SBS modified asphalt under 70 °C wet dry cycle aging. This indicates that the degradation degree of SBS modified asphalt is intensified under 70 °C conditions, which seriously affects the low-temperature ductility of modified asphalt.

### 3.3 Effects of different aging methods on the high temperature performance of SBS modified asphalt

**Fig 6:**
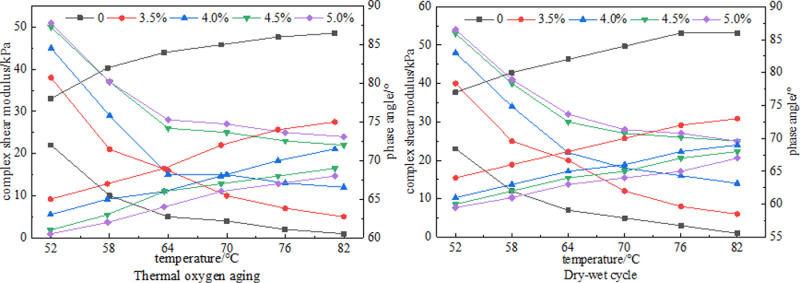
DSR test results.

SBS modified asphalt with different dosages was subjected to short-term aging under different aging conditions. The aged samples were temperature scanned through DSR test to obtain the complex shear modulus G* and phase angle of the samples, which were used to characterize the changes in mechanical properties of SBS modified asphalt with different dosages after aging. The results are shown in Fig 6.

As shown in Fig 6, the changes in complex shear modulus and phase angle of SBS modified asphalt with different dosages after short-term aging are presented. The complex shear modulus decreases overall with increasing temperature, while the phase angle increases with increasing temperature. The increase in SBS content leads to an increase in complex shear modulus and a decrease in phase angle. Both thermal oxidative aging and wet dry cycle aging follow the same pattern. The decrease in complex shear modulus under thermal oxidative aging conditions is higher than that under dry wet cycle aging. Under thermal oxidative aging conditions, the matrix asphalt and SBS modified asphalt with different dosages decrease by 95.5%, 86.8%, 73.3%, 56.0%, and 52.0%, respectively. After dry wet cycle aging, the matrix asphalt and SBS modified asphalt with different dosages decrease by 95.0%, 85.0%, 70.8%, 52.8%, and 52.0%, respectively. The decrease in complex shear modulus under dry wet cycle aging conditions is smaller than that under thermal oxidative aging. Analysis suggests that thermal oxidative aging directly damages the molecular structure of asphalt and SBS, while dry wet cycling is mainly characterized by local physical damage. Therefore, the impact of thermal oxidative aging on G * is more significant.Under thermal oxidative aging conditions, the phase angles of matrix asphalt and SBS modified asphalt with different dosages increased by 10.9%, 15.4%, 13.5%, 13.1%, and 12.4%, respectively. Under wet dry cycling conditions, the phase angles of matrix asphalt and SBS modified asphalt with different dosages increased by 13.0%, 14.0%, 13.0%, 13.0%, and 12.0%, respectively. Overall, compared to the base asphalt (SBS content of 0), the high-temperature performance of SBS modified asphalt has been improved. Under thermal oxidative aging conditions, the decrease in complex shear modulus is greater than that under wet dry cycles. It can be considered that thermal oxidative aging has a greater impact on the deformation resistance of SBS asphalt.

**Fig 7:**
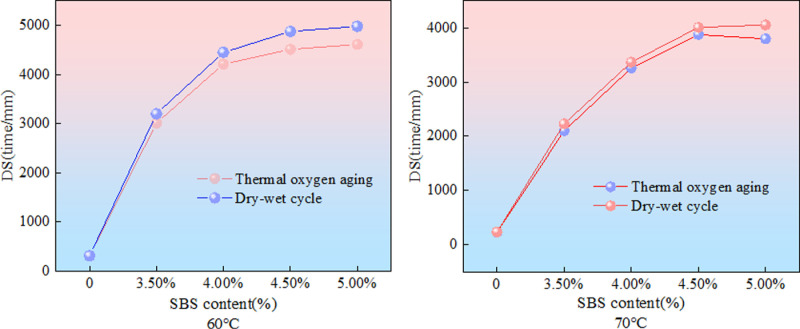
Results of rutting test.

The specimens of SBS modified asphalt mixtures with different dosages were subjected to thermal oxidative aging and wet dry cycle aging, and then the dynamic stability of SBS modified asphalt mixtures with different dosages after different aging methods was obtained through rutting tests. Due to the high road surface temperature of up to 70 °C in hot and humid areas during summer, the rutting test was conducted in environments of 60 °C and 70 °C, respectively. The test results are shown in Fig 7.

As shown in Fig 7, the rutting test results of aged SBS asphalt mixture with different dosages show that the dynamic stability of SBS modified asphalt mixture is significantly better than that of matrix asphalt mixture, and the dynamic stability gradually increases with the increase of SBS content. For SBS asphalt mixture, the dynamic stability increased by 53.3% and 55.6% at 60 °C, and 80.9% and 81.9% at 70 °C. The growth rate of dynamic stability at 70 °C was significantly higher than that at 60 °C, indicating that SBS effectively improved the anti rutting performance of asphalt mixture. However, the dynamic stability at 70 °C was lower than that at 60 °C. Overall, the increase in dynamic stability after wet dry cycle aging is greater than that after thermal oxidative aging, indicating that thermal oxidative aging has a greater impact on the high-temperature stability of SBS asphalt mixtures.

## 4. Conclusion

Based on the unique climatic features of high-temperature and humid regions, this article simulates such conditions through indoor experiments to investigate the performance degradation patterns of SBS modified asphalt under various conditions. It conducts a macro perspective study on the performance indicators of SBS modified asphalt, considering different dosages of SBS modified asphalt and the impact of various aging conditions, temperatures, and aging durations. Through experimental research and data analysis, the following conclusions were drawn:

(1) As aging time increases, whether through thermal oxidative aging or wet-dry cycle aging, the penetration ratio and ductility ratio of SBS modified asphalt with varying dosages decrease, while the softening point ratio increases. This is due to the accumulation of time, ultimately leading to intensified aging of SBS modified asphalt and reduced stability. Under wet-dry cycling conditions, the change rates of the three indicators for SBS modified asphalt with a dosage of 4.5% are all less than those for SBS modified asphalt with a dosage of 4.0%. Under thermal oxidative aging conditions, the modified asphalt with a dosage of 4.5% demonstrates greater stability.
(2) The impact of temperature on the aging process of SBS modified asphalt cannot be overlooked. The changes in penetration ratio, softening point ratio, and ductility ratio after aging at 70°C are significantly greater than those at 60°C. Furthermore, during wet-dry cycle aging, an increase in the dosage of SBS modifier slows down the decay rate of the penetration ratio, softening point ratio, and ductility ratio of SBS modified asphalt.
(3) In terms of high-temperature performance, thermal-oxidative aging exhibited a more pronounced impact on SBS-modified asphalt compared to wet-dry cycle aging. Based on comprehensive analysis of performance variations in SBS-modified asphalt with different additive contents after aging under both conditions, the asphalt with 4.5% SBS content demonstrated relatively stable changes in the three key parameters (penetration, ductility, and softening point) and high-temperature performance. It was concluded that a 4.5% SBS dosage is optimal for asphalt pavements in high-temperature and high-humidity regions, ensuring sufficient moisture resistance to prevent accelerated aging caused by water infiltration.

For future work, experimental protocols were designed to simulate the complex multi-factor coupled environments typical of hot and humid regions (e.g., temperature fluctuations, prolonged high humidity, and UV intensity variations). These efforts aimed to reveal aging patterns under synergistic multi-factor interactions, establish more precise accelerated aging test standards, and build a long-term performance database for SBS-modified asphalt in hygrothermal environments. The data will support the optimization of lifespan prediction models and clarify quantitative relationships between “microstructural degradation → macroscopic performance decline,” thereby guiding the targeted development of anti-aging additives. This approach is expected to enhance the durability of SBS-modified asphalt in hygrothermal conditions and extend pavement service life.

## Supporting information

S1 Data Raw data. (XLSX)

S2 Data Raw data (XLSX)
